# Comparison of 3D printed prostate models with standard radiological information to aid understanding of the precise location of prostate cancer: A construct validation study

**DOI:** 10.1371/journal.pone.0199477

**Published:** 2018-06-25

**Authors:** Jan Ebbing, Fredrik Jäderling, Justin W. Collins, Olof Akre, Stefan Carlsson, Jonas Höijer, Mats J. Olsson, Peter N. Wiklund

**Affiliations:** 1 University Hospital Basel, Urological University Clinic Basel-Liestal, Basel, Switzerland; 2 Karolinska University Hospital, Division of Urology, Stockholm, Sweden; 3 Karolinska University Hospital, Division of Radiology, Stockholm, Sweden; 4 Karolinska Institutet, Department of Molecular Medicine and Surgery (MMK), Stockholm, Sweden; 5 Karolinska Institutet, Unit of Biostatistics, Institute of Environmental Medicine (IMM), Stockholm, Sweden; University of South Alabama Mitchell Cancer Institute, UNITED STATES

## Abstract

**Background:**

To investigate the reliability with which healthcare professionals with different levels of expertise are able to impart the exact location of prostate cancer (PCA) after (A) reading written magnetic resonance imaging (MRI) reports, (B) attending MRI presentations in multidisciplinary team meetings (MDT), and (C) examining 3D printed prostate models, which represents a new technology to describe the location of PCA lesions.

**Methods:**

We used three different PCA cases to assess the three information tools. Construct validation was performed using two healthcare groups with different levels of expertise: (1) Nine expert urologists in PCA, and (2) nine medical students. After each information tool, the study participants plotted the tumor location in a 2-dimensional prostate diagram. A scoring system was established to evaluate the drawings in terms of accuracy of plotting tumor position. Data are shown as median scores with interquartile range.

**Results:**

Within the expert group, no significant difference was seen in the overall scoring results between the information tools (p = 0.34). Medical students performed significantly worse with MDT information (p = 0.03). Experts performed better in all three information tools compared to students, resulting in a significantly 25% higher overall total score (25.0[22.3–26.7] vs. 20.0[15.0–24.0], p<0.001). The difference was largest after MDT information, with experts showing a 49% better scoring (p<0.001), and second largest with the 3D printed models, showing a 17% better scoring of the experts (p = 0.07). No difference was found in the written MRI report scoring results between experts and students.

**Conclusions:**

3D printed models provided better orientation guide to medical students compared to MDT MRI presentations. This indicates that the 3D printed models might be easier to understand than the current gold standard MDT conferences. Therefore, 3D models may play an increasingly important role in providing guidance for orientation for less experienced individuals, such as surgical trainees.

## Introduction

The precise knowledge of the three-dimensional (3D) location of a prostate cancer and its proximity to the prostate capsule and neurovascular bundle has implications for surgical therapeutic strategies, which in turn can influence both functional and oncological outcomes [1–5]. Apart from prostate biopsy results and the digital-transrectal prostate palpation, magnetic resonance imaging (MRI) written reports, and multidisciplinary team meetings (MDT), presenting two-dimensional images obtained from MRI, are current standard methods to describe the location of prostate cancer (PCA), to transmit the information to health care professionals, and to orientate them for surgical planning. 3D printed models are a novel orientation tool, which has recently gained much interest in the medical world. First proof of concept studies, investigating the use of customized, patient-specific printed 3D models of the prostate and cancer lesions to aid in prostate cancer surgery, have been published [6,7]. However, the reliability of all three different information tools to impart the exact location of prostate cancer in the prostate to healthcare professionals is unknown.

This is the first study to investigate how accurately the exact location of a single prostate cancer lesion can be assessed by medical personal with different experience-levels. The tools that are currently used to transfer knowledge include MRI written reports and MRI exams presented in MDT. Patient-specific 3D printed prostate models represent a novel and promising technology and information tool that can be employed in the management of prostate cancer [8].

## Materials and methods

The study was planned and performed at Karolinska University Hospital and Karolinska Institutet, Stockholm, Sweden. The study protocol was in accordance with the regulations of the local Ethics Committee of the Karolinska Institutet which approved the study, and written informed consent was obtained from all study participants. In order to avoid biases resulting from learning effects over time, we used a 3x3 Latin square array with three different PCA cases to assess the three information tools for the orientation of prostate cancer locations: (A) Written MRI report, (B) 3D printed model, and (C) MRI presentation in MDT. Tumor characteristics were:

1. cT2, PI-RADS 5 (size 15x9x21 mm), mriT3, pT3a, Gleason 4+3 (tertiary grade 5) for case 1;
2. cT2-3, PI-RADS 5 (size 19x12x17 mm), mriT3, pT2c, Gleason 4+3 for case 2;
3. T1c, PI-RADS 5 (size 27x11x21 mm), mriT3, pT2c, Gleason 3+3 (tertiary grade 4) for case 3.

The case records were obtained anonymously; therefore, the Ethic Committee of the Karolinska Institutet has waived the need for obtaining written informed consent of the three patients. We aimed at achieving adequate construct validation by using two medical groups with different levels of expertise: (1) Expert urologists in PCA, and (2) Medical students. The fact that the capability of 3D prostate models in assisting the orientation of PCA location among people with different levels of medical expertise is mostly unknown led to the decision to compare these two groups with medical background, but with a high difference in PCA exposure. Expert urologists in PCA were chosen by means of their experience in prostate cancer surgery, with at least 200 performed radical prostatectomies and ongoing regular activity in performing radical prostatectomies. In addition, all participants in the expert group were console surgeons for robot-assisted radical prostatectomies and recruited from Karolinska University Hospital, Stockholm, Sweden. All medical students were studying at Karolinska Institutet Medical University, Stockholm, Sweden. The majority of medical students has had exposure to urological surgical training and had observed at least one radical prostatectomy before participating in the study. None of the medical students had actively assisted in a radical prostatectomy. We included nine participants in each group. The three information tools were presented according to the 3x3 Latin square array in the tool orders ABC, BCA, or CAB; each order was presented to three participants of each group. All three different PCA cases were presented consecutively in a different case order in each information tool (tool A—case order: 2, 1, 3; tool B—case order: 3, 1, 2; tool C—case order: 1, 2, 3), before the next information tool was presented to the participants. All information tools were presented in a time period of less than 48 hours. The same nomenclature was used when describing tumor locations in the written MRI reports and at the MDT. MRI presentation in MDT was performed one time for each case in a structured and standardized way showing and explaining the tumor location and dimension in axial, coronal and sagittal planes. Participants were allowed to ask for one repeated, immediate, standardized presentation. All MRI presentations in MDT were performed by the same radiologist, who was highly experienced in radiological PCA diagnostics. The participants were allowed to read the written MRI reports repeatedly. The 3D printed prostate models had no additional labels or markings to orientate the specimen. The participants were allowed to examine the 3D printed models by hand. After each presented case, the study participants were asked to plot the tumor location in a 2-dimensional prostate diagram (Swedish national biopsy and MRI report template). Capsular invasion was not a criterion to be judged by the participants. No time limit was set to plot the tumor location. A scoring system was used to evaluate the accuracy of tumor position in the drawings, with maximum achievable points of 30 per case.

### Scoring system

**Fig 1:**
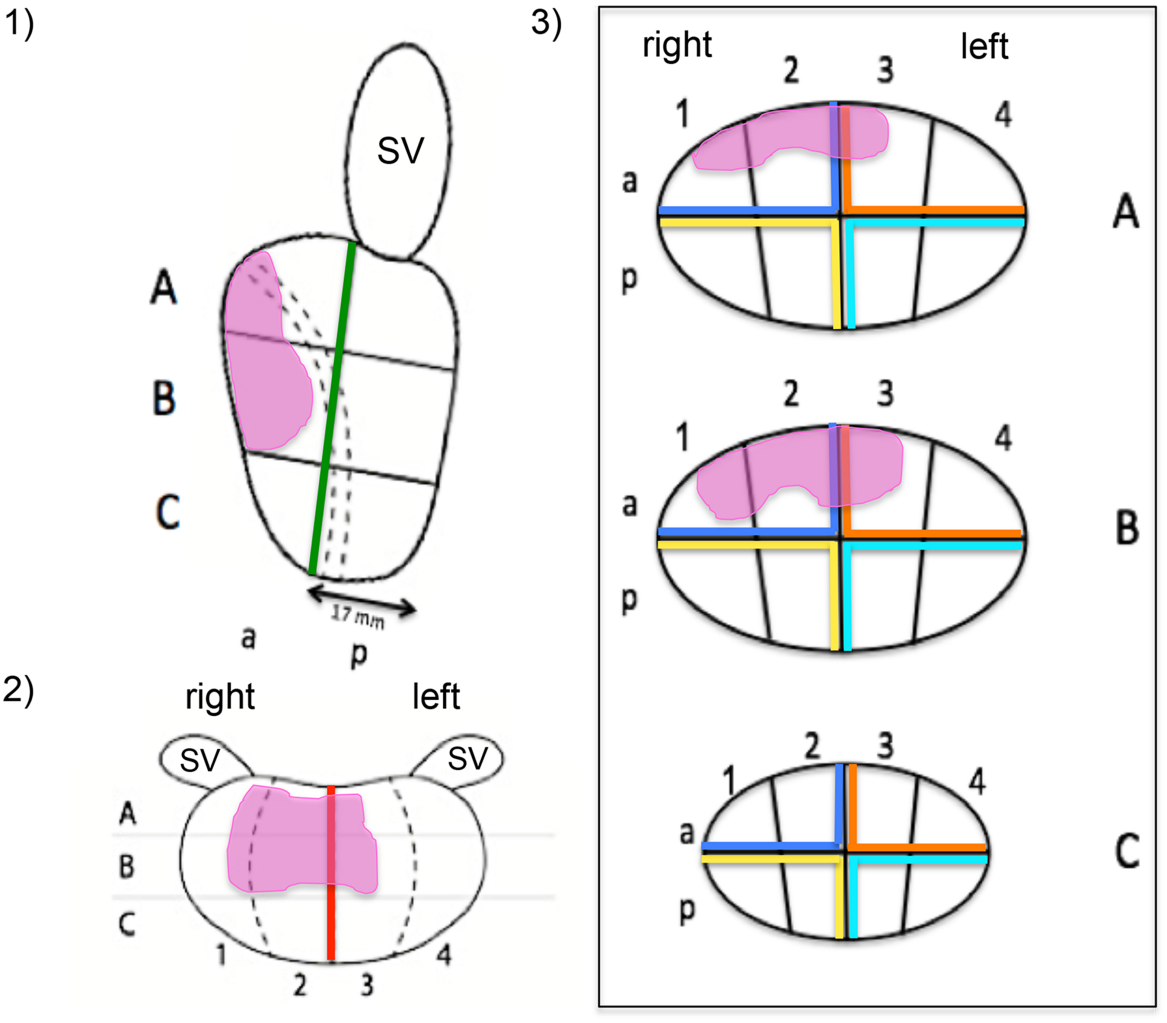
Swedish national prostate biopsy and MRI report template. 1) Side (sagittal) view of the prostate, 2) ventral (coronal) view of the prostate, 3) transverse (axial) sections A-C of pictures 1) and 2). The study participants used this diagram without the colour lines to plot the prostate tumor location. The correctly plotted tumor location of case 3 is shown in the diagram (pink areas). For the scoring system picture 1) was divided in an anterior and posterior half (green line), picture 2) in a right and left half (red line) and picture 3 A-C in quadrants (blue, orange, yellow, turquoise). (MRI) magnetic resonance imaging; (SV) seminal vesicle; (a) anterior; (p) posterior; broken lines in picture 1) mark the urethra.

After an extensive literature review, no suitable validated scoring system could be identified that measures the accuracy of displaying a tumor location on a 2-dimensional diagram. Therefore, we developed our own scoring system, which was adapted to the Swedish national biopsy and MRI report template (Fig 1). The prostate diagram consisted of a plotted side (sagittal) view of a prostate (Fig 1, picture 1), a ventral (coronal) view (Fig 1, picture 2), and three transverse (axial) sections A-C corresponding to pictures 1 and 2 (Fig 1, picture 3). The maximum scoring for each picture (1, 2, 3A, 3B, and 3C) was 10 points, respectively. The sum of points for pictures 3A-3C was divided by three to get the total points for the whole picture 3, resulting in a maximum total score of 30 points for a 100% correctly plotted tumor location in the entire prostate diagram. The scoring system was designed to allow the detection of major and minor mistakes in plotting of the tumor location. Plotting the tumor mainly (> 50%) in the correct half in picture 1 (anterior or posterior) or 2 (right side or left side) or mainly (> 50%) in the correct quarter in pictures 3A-3C was each scored with 10 points. A plot mainly in the wrong half or quarter resulted in 0 points for the picture, considering wrong “anterior-posterior” and “right-left” plots or wrong plots in main quadrants as major mistakes. If 10 points for a picture were scored, points were deducted in case of minor mistakes in tumor location and tumor size. For each incorrectly marked box (tumor marked in a non-tumor-box, or tumor not marked in a tumor-box, or tumor marked in a tumor-box but with incorrect size (marked with > 50% of the box although it should be marked with < 50% or marked with > 50% although it should be marked with < 50%)), one point was deducted. In a box that should be marked with exactly 50% of the full plain, a point was deducted in case of a tumor that was plotted in the box with either < 25% or > 75% of the plain.

### Construct validation of the scoring system

We performed construct validation of our scoring system by both comparing the two groups of expertise (group 1 –expert urologists and group 2 –medical students) within the two standard information tools “A—Written MRI report” and “C—MRI presentation in MDT” and by comparing group 1 and group 2 both within information tool A and C.

Using two established information tools of different complexity ensures construct validation of our self-designed new scoring system adapted to the Swedish national biopsy and MRI report template. The written MRI report is standardized and therefore easier to understand [9–11]. The MRI presentation in MDT represents a very complex information standard that transfers information of different quality, e.g. visually and acoustically, at the same time [9–11]. This kind of construct validation process to proof validity of a new measure is well-established and scientifically accepted [12].

Due to the different grade of complexity of the two information tools, we hypothesized that the differences in the scoring results between expert urologists and medical students would be smaller in the written MRI reports and larger in the MRI presentations in MDT. In addition, we assumed that medical students would perform better with the written MRI reports compared to the MRI presentations in MDT and that no difference between the two information tools would be found in the group of expert urologists, thus emphasizing their expert status [13,14].

### Magnetic resonance imaging (MRI)

A 3-Tesla scanner (Magnetom Verio, Siemens Medical Systems, Erlangen, Germany) with a 32-channel phased array pelvic coil was used to scan the prostate. The MRI protocol presented at the MDT conference was bi-parametric. The protocol included: T2-weighted (T2w) sagittal and axial (transverse) acquisition, coronal 3-dimensional (3D) T2w TSE SPACE (sampling perfection with application optimized contrasts using different flip angle evolution) with isotropic voxels (0.8 × 0.8 × 0.8 mm), axial diffusion weighted imaging (DWI) RESOLVE (readout segmentation of long variable echo-trains) b-values 50, 200, 1000 with computed b 1500, and apparent diffusion coefficient (ADC) map, as well as axial T1w TSE covering the small pelvis from the aortic bifurcation through the whole pelvis. The Prostate Imaging and Report and Data System—Version 2 (PI-RADS v2) was used for the interpretation and reporting of the prostate MRI examinations [10,11].

### Transfer of MRI data to the 3D printed model

**Fig 2:**
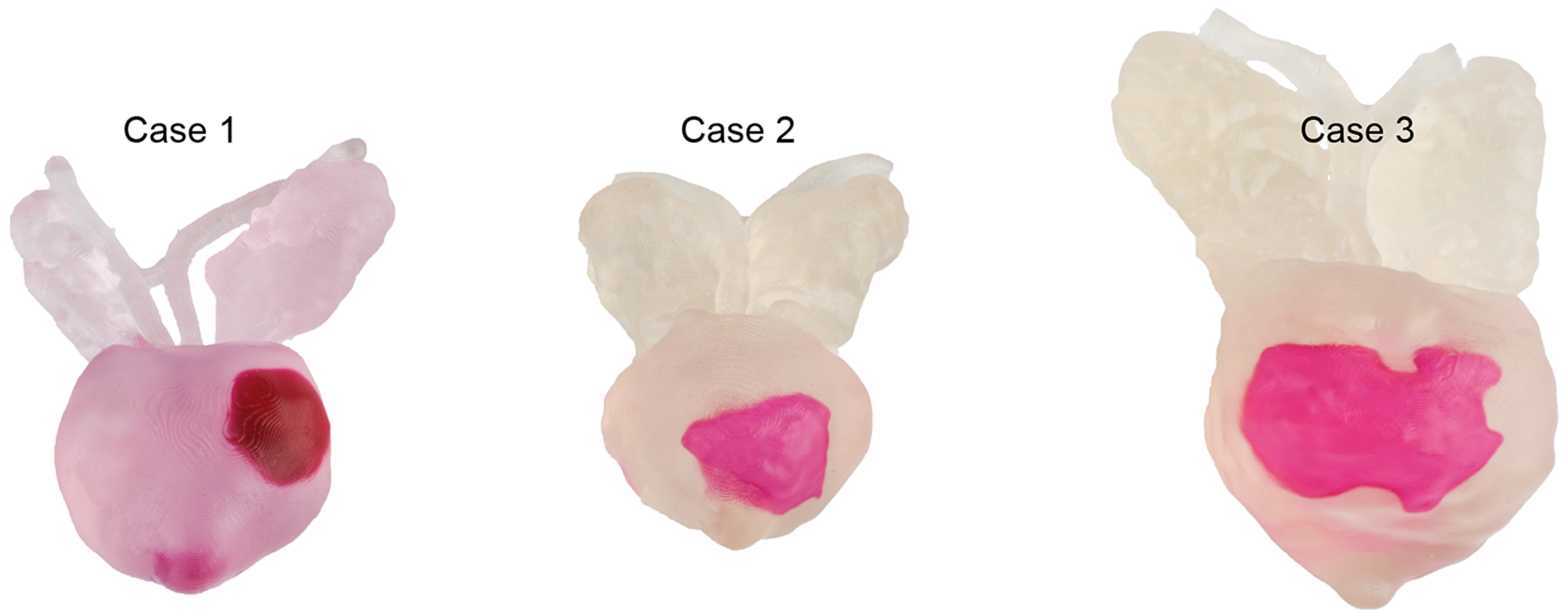
3D printed prostate models (case 1–3) used in the study. The models were manufactured by 3D Systems, Inc. 5381 South Alkire Circle, Littleton, Colorado 80127 USA. Each prostate model contained a single prostate cancer lesion (pink colour). Tumor characteristics for case 1 were cT2, PI-RADS 5 (size 15x9x21 mm), mriT3, pT3a, Gleason 4+3 (tertiary grade 5); for case 2 cT2-3, PI-RADS 5 (size 19x12x17 mm), mriT3, pT2c, Gleason 4+3; and for case 3 T1c, PI-RADS 5 (size 27x11x21 mm), mriT3, pT2c, Gleason 3+3 (tertiary grade 4).

**Fig 3:**
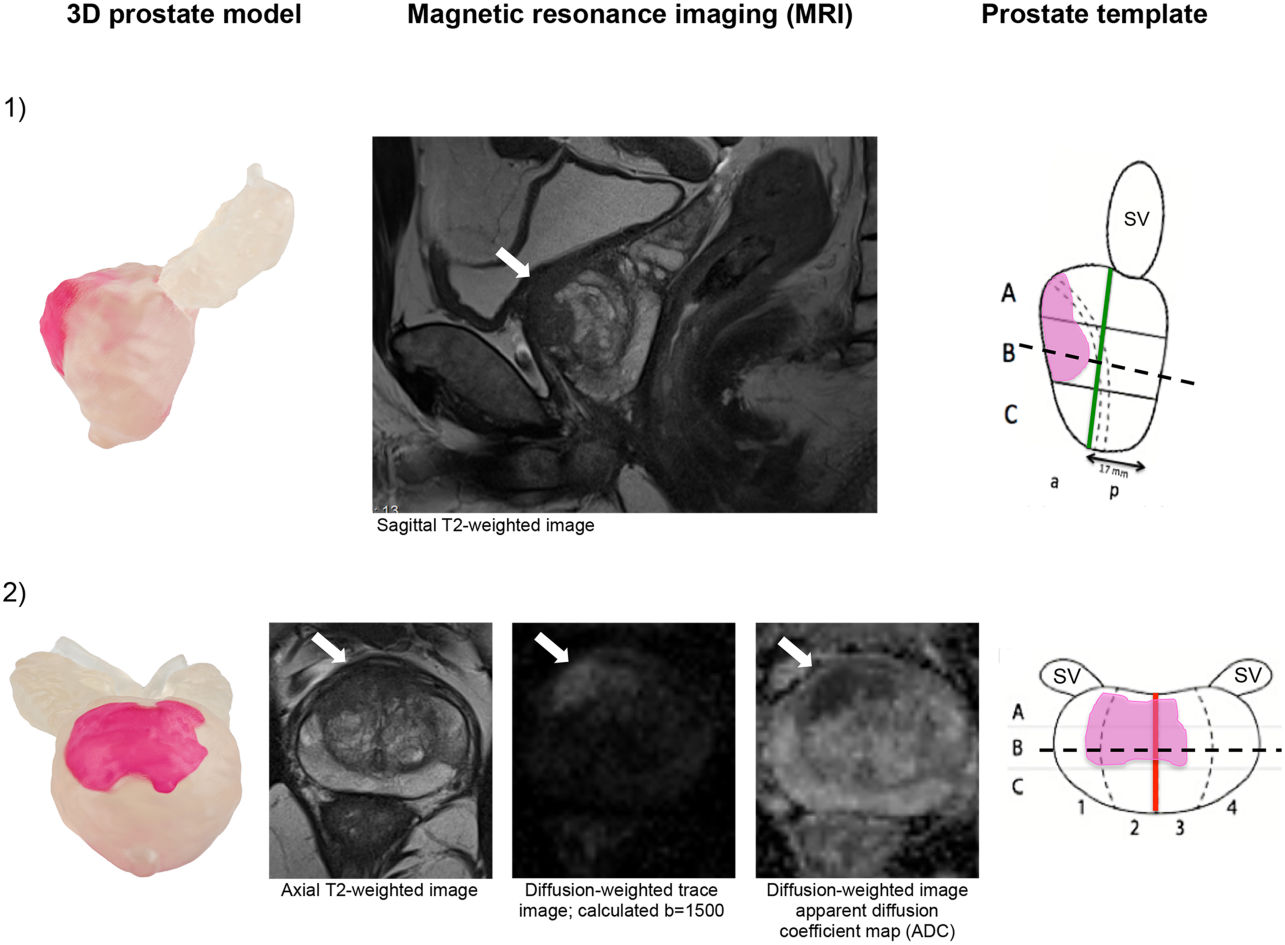
Prostate cancer lesion of case 3 according to 3D printed prostate model, MRI and prostate template. In (1) corresponding side (sagittal) views and (2) according to ventral (coronal) views in 3D printed prostate model and prostate template and different sequences of the corresponding axial view in MRI (corresponds to broken line in section B). (SV) seminal vesicle, (a) anterior, (p) posterior. Arrows indicate prostate cancer in MRI.

The three 3D printed prostate models (Fig 2) were produced from axial reconstructions of the coronal 3D T2w MRI sequence (SPACE) with a slice thickness of 0.8 mm. The prostatic surface as well as each tumor was delineated in each axial slice, carried out in Microsoft power point. The delineation map was transferred to the 3D printing equipment to create the printed 3D prostate model. Fig 3 depicts the prostate cancer lesion of case 3 in different views for the 3D printed prostate model, the corresponding MRI, as well as the correctly performed drawing in the prostate diagram.

### Statistical analysis

Data are shown as median scores with interquartile range (IQR) or as proportions. To examine whether there were any differences in median scores between either the two groups of participants or the three information tools, median regression was used. Since each participant evaluated more than one case, cluster robust standard errors were used to account for dependency in the data [15]. To analyze potential differences between the two groups concerning proportions of major mistakes and complete major accuracy, logistic regression with cluster robust standard errors was used. In addition, to underline construct validity of the scoring system, Spearman’s correlation test was applied to determine the association between the extent of expertise (expert urologists versus medical students) and the overall total score achieved in each information tool.

## Results

### Construct validity of the scoring system

Our scoring system evaluated the performance of the expert urologists in the MRI presentations in MDT statistically better compared to the performance of the medical students (25.3 [23.7–27.0] vs. 17.0 [12.3–21.7], p < 0.001). Experts scored also slightly better with the MRI written reports compared to the medical students, but this difference was not statistically significant (24.0 [22.0–26.0] vs. 22.0 [17.0–24.7], p = 0.25). Correspondingly, Spearman´s correlation revealed a significant association between the overall scoring result and the level of expertise (medical students vs. expert urologists) in MRI presentations in MDT (correlation coefficient: r = 0.61 [95% CI 0.41–0.75], p < 0.001), and a borderline significance in the written MRI reports (correlation coefficient: r = 0.25 [95% CI -0.02–0.48], p = 0.07). In addition, in both information tools, the inter quartile ranges of the scoring results of the medical students were much greater compared to the expert group, underlining construct validity of the scoring system. As hypothesized, the medical students performed significantly better with the MRI written reports compared to the MRI presentations in MDT (median regression coefficient: r = -5.0 [95% CI -8.81–-1.19], p = 0.01), whereas no significant difference was found in the expert group (median regression coefficient: r = 1.33 [95% CI -0.49–3.16], p = 0.15).

In conclusion, construct validity of our self-designed scoring system was achieved. The scoring system proved to be able to discriminate between both different levels of expertise and between information tools of different complexity.

### Construct validity of the 3D printed prostate models compared to MRI written reports and MRI presentations in MDT

A total of 162 prostate diagram drawings (2 groups of different expertise—each with 9 participants x 3 information tools x 3 PCA cases), including the results from the construct validation of the scoring system, were evaluated to investigate the significance of the 3D printed prostate models compared to the written MRI reports and MRI presentations in MDT to impart the exact location of PCA by healthcare professionals of different expertise. The overall median total score and the scores sub-classified for information tool A (written MRI report), B (3D printed prostate model), and C (MRI presentation in MDT) in group 1 (expert urologists) and group 2 (medical students) are summarized in Table 1. With 25.3 (23.7–27.0) of 30 reachable points, the experts showed the best performance in information tool C (MRI presentation in MDT), followed by tool B (3D printed prostate model; 25.0 [20.0–26.7] points) and tool A (written MRI report; 24.0 [22.0–26.0] points). However, these differences were statistically non-significant (p = 0.34). When compared with tools A (written MRI report; 22.0 [17.0–24.7] points) and tool B (3D printed prostate model; 21.3 [17.3–23.7] points), medical students performed significantly worse with tool C (MRI presentation in MDT; 17.0 [12.3–21.7] points; p = 0.03). Experts performed better in all three information tools compared to students, resulting in a significantly higher overall total score in the expert group (25.0 [22.3–26.7] versus 20.0 [15.0–24.0], p<0.001). The difference was greatest and statistically significant for tool C (MRI presentation in MDT; 25.3 [23.7–27.0] versus 17.0 [12.3–21.7]; p<0.001).

**Table 1:** Overall total score (median with IQR) and the scores sub-classified for information tool A (written MRI report), information tool B (3D printed model) and information tool C (MRI presentation in MDT) in group 1 (expert urologists) and group 2 (medical students).

	Information tool A (Written MRI report)	Information tool B (3D printed model)	Information tool C (MRI presentation in MDT)	Overall total score	p-value
**Group 1** Expert urologists	24.0 (22.0–26.0) 80.0%	25.0 (20.0–26.7) 83.3%	25.3 (23.7–27.0) 84.3%	25.0 (22.3–26.7) 83.3%	0.34
**Group 2** Medical students	22.0 (17.0–24.7) 73.3%	21.3 (17.3–23.7) 71.0%	17.0 (12.3–21.7) 56.6%	20.0 (15.0–24.0) 66.7%	0.03
**p-value**	0.25	0.07	<0.001	<0.001	

Data also show the results in percentage from the maximum achievable score of 30 points (100%). (IQR) interquartile range.

Table 2 shows the scoring results for group 1 (expert urologists) and group 2 (medical students), sub-classified for the different views (side, ventral, and overall transverse view (ABC)) of the prostate diagram, with a maximum of 10 possible points for each view. Concerning all 81 drawings (overall scoring) of group 1 and 2, respectively, the experts performed better in all three views, with the greatest difference in the overall transverse view (9.0 [7.7–9.3] versus 6.3 [3.3–8.7], p < 0.001).

**Table 2:** Scoring results (median with IQR) for group 1 (expert urologists) and group 2 (medical students), sub-classified for pictures 1–3 of the prostate template (side (sagittal) view, ventral (coronal) view, overall transverse (axial) view (ABC)) (s. Fig 1) in information tool A (written MRI report), information tool B (3D printed model) and information tool C (MRI presentation in MDT).

	Group 1 Expert urologists	Group 2 Medical students	p-value
**Overall scoring**			
(all information tools, all cases)	25 (22.3–26.7)	20.0 (15.0–24.0)	< 0.001
Side (sagittal) view	9.0 (8.0–9.0)	8.0 (6.0–9.0)	0.20
Ventral (coronal) view	8.0 (7.0–9.0)	7.0 (5.0–8.0)	0.35
Overall transverse (axial) view (ABC)	9.0 (7.7–9.3)	6.3 (3.3–8.7)	< 0.001
**Information tool A**			
Written MRI reports (all cases)	24.0 (22.0–26.0)	22.0 (17.0–24.7)	0.25
Side (sagittal) view	8.0 (8.0–9.0)	8.0 (6.0–9.0)	1.0
Ventral (coronal) view	8.0 (7.0–9.0)	7.0 (6.0–8.0)	0.06
Overall transverse (axial) view (ABC)	8.7 (7.7–9.0)	8.3 (3.3–9.0)	0.52
**Information tool B**			
3D printed models (all cases)	25.0 (20.0–26.7)	21.3 (17.3–23.7)	0.07
Side (sagittal) view	9.0 (8.0–10.0)	9.0 (8.0–9.0)	1.0
Ventral (coronal) view	8.0 (6.0–9.0)	7.0 (5.0–8.0)	0.04
Overall transverse (axial) view (ABC)	8.3 (5.0–9.0)	6.3 (3.3–8.3)	0.07
**Information tool C**			
MRI presentations in MDT (all cases)	25.3 (23.7–27.0)	17.0 (12.3–21.7)	< 0.001
Side (sagittal) view	9.0 (8.0–9.0)	8.0 (0.0–9.0)	0.08
Ventral (coronal) view	8.0 (7.0–9.0)	7.0 (4.0–8.0)	0.09
Overall transverse (axial) view (ABC)	9.3 (8.7–9.7)	5.7 (3.3–8.7)	< 0.001

(IQR) interquartile range.

Looking at the information tools separately, this difference is mainly explained by worse performances of the medical students in the ventral and overall transverse view of the 3D printed models and in particular in the overall transverse view during MRI presentations in MDT. No significant differences were detected for the different views in the written MRI reports between experts and medical students.

The overall number of major mistakes was higher and the rates of complete major accuracy (0% rate of major mistakes) was lower in the medical students group compared to the experts in all three information tools (Table 3). Notably, the rates of major mistakes (Table 3A) and of complete major accuracy (Table 3B) are differently distributed between the three information tools in the expert and medical student group. Whilst in the expert group, the major mistake rate was highest in the 3D printed models (15.6%) and lowest in the MRI presentations in MDT (3.0%), in the medical students group, the major mistake rates were equally high in the 3D printed models (27.4%) and in the MRI presentations in MDT (26.7%), but lower in the written MRI reports (16.3%). According to this, the accuracy was highest in MRI presentations in MDT (88.8%) and lowest in the 3D printed models (63.0%) in the expert group. In the medical students group, accuracy was higher in the written MRI reports (55.6%) compared to the two other tools.

**Table 3:** Summarized rates (%) of (A) major mistakes (s. Table 4A1) and (B) rates (%) of complete major accuracy (0% major mistakes) (s. Table 4A2) in group 1 (expert urologists) and group 2 (medical students) subdivided for information tools A-C.

	Group 1 Expert urologists	Group 2 Medical students	p-value
**(A) Rates of major mistakes**			
**Information tool A** (Written MRI reports)	8.9%	16.3%	0.38
**Information tool B** (3D printed models)	15.6%	27.4%	0.17
**Information tool C** (MRI presentations in MDT)	3.0%	26.7%	< 0.001
**(B) Rates of complete major accuracy**			
**Information tool A** (Written MRI reports)	81.5%	55.6%	0.14
**Information tool B** (3D printed models)	63.0%	25.9%	0.02
**Information tool C** (MRI presentations in MDT)	88.8%	29.6%	< 0.001

On closer inspection of major and minor mistakes (Table 4), the reasons for the worse scoring of the students in the 3D printed models, but especially in the MRI presentations in MDT, become more obvious. The medical students tended to make more major mistakes in the transverse views in the 3D printed prostate models, and particularly in the MRI presentations in MDT, in which the differences became even statistically significant, p = 0.01. In addition, the sagittal (side) view in the MRI presentations in MDT seemed to be a major mistake issue (decision between anterior and posterior location of the tumor) for the students (Table 4A1).

**Table 4:** (A1) rates (%) of major mistakes (total loss of 10 points) in 135 judgments (9 participants x 3 PCA cases x 5 views to judge/case = 135) sub-classified for the five different views in pictures 1–3 of the prostate template (Fig 1) and subdivided for group 1 (Expert urologist) and group 2 (Medical students), and (A2) rates of complete major accuracy (0% major mistakes) out of 27 judgments (9 participants x 3 PCA cases) in the experts and medical students group subdivided for information tool A (Written MRI report), information tool B (3D printed model) and information tool C (MRI presentation in MDT) respectively. (B) minus points (median with IQR) for minor mistakes in the experts und medical students group, sub-classified by (B1) type of minor mistake and by (B2) different views in pictures 1–3 of the prostate template (side (sagittal) view, ventral (coronal) view, transverse (axial) views (ABC)) in information tool A (Written MRI report), information tool B (3D printed model) and information tool C (MRI presentation in MDT).

	Group 1 Expert urologist	Group 2 Medical students	p-value
**A1** **Rate of major mistakes (total loss of 10 points) in all presented cases sub-classified by the five different views in pictures 1–3 of the prostate template**			
**Information tool A—Written MRI reports** (all cases)			
Overall	12/135 (8.9%)	22/135 (16.3%)	
Side (sagittal) view	2/27 (7.4%)	3/27 (11.1%)	1.00
Ventral (coronal) view	0/27 (0.0%)	0/27 (0.0%)	1.00
Transverse (axial) view A	4/27 (14.8%)	9/27 (33.3%)	0.20
Transverse (axial) view B	4/27 (14.8%)	5/27 (18.5%)	1.00
Transverse (axial) view C	2/27 (7.4%)	5/27 (18.5%)	0.42
**Information tool B—3D printed models** (all cases)			
Overall	21/135 (15.6%)	37/135 (27.4%)	
Side (sagittal) view	4/27 (14.8%)	2/27 (7.4%)	0.67
Ventral (coronal) view	2/27 (7.4%)	3/27 (11.1%)	1.00
Transverse (axial) view A	5/27 (18.5%)	9/27 (33.3%)	0.35
Transverse (axial) view B	6/27 (22.2%)	12/27 (44.4%)	0.15
Transverse (axial) view C	4/27 (14.8%)	11/27 (40.7%)	0.07
**Information tool C—MRI presentations in MDT** (all cases)			
Overall	4/27 (3.0)	36/135 (26.7%)	
Side (sagittal) view	0/27 (0.0%)	7/27 (25.9%)	0.01
Ventral (coronal) view	1/27 (3.7%)	2/27 (7.4%)	1.00
Transverse (axial) view A	1/27 (3.7%)	9/27 (33.3%)	0.01
Transverse (axial) view B	1/27 (3.7%)	9/27 (33.3%)	0.01
Transverse (axial) view C	1/27 (3.7%)	9/27 (33.3%)	0.01
**A2** **Rate of major mistakes in all presented cases sub-classified by ratios (%) of major mistakes per case plotted in pictures 1–3 of the prostate template**			
**Information tool A—Written MRI reports** (all cases)			0.38
0% mistakes (no major mistake)	22/27 (81.5%)	15/27 (55.6%)	
20% mistakes	0/27 (0.0%)	4/27 (14.8%)	
40% mistakes	3/27 (11.1%)	6/27 (22.2%)	
60% mistakes	2/27 (7.4%)	2/27 (7.4%)	
80% mistakes	0/27 (0.0%)	0/27 (0.0%)	
100% mistakes (max. of 5 major mistakes)	0/27 (0.0%)	0/27 (0.0%)	
**Information tool B– 3D printed models** (all cases)			0.17
0% mistakes (no major mistake)	17/27 (63.0%)	7/27 (25.9%)	
20% mistakes	5/27 (18.5%)	11/27 (40.7%)	
40% mistakes	1/27 (3.7%)	3/27(11.1%)	
60% mistakes	2/27 (7.4%)	4/27 (14.8%)	
80% mistakes	2/27 (7.4%)	2/27 (7.4%)	
100% mistakes (max. of 5 major mistakes)	0/27 (0.0%)	0/27 (0.0%)	
**Information tool C—MRI presentations in MDT** (all cases)			< 0.001
0% mistakes (no major mistake)	24/27 (88.9%)	8/27 (29.6%)	
20% mistakes	2/27 (7.4%)	7/27 (25.9%)	
40% mistakes	1/27 (3.7%)	7/27 (25.9%)	
60% mistakes	0/27 (0.0%)	5/27 (18.5%)	
80% mistakes	0/27 (0.0%)	0/27 (0.0%)	
100% mistakes (max. of 5 major mistakes)	0/27 (0.0%)	0/27 (0.0%)	
**B1** **Minor mistakes sub-classified by type of minor mistake**			
**Information tool A—Written MRI reports** (all cases)			
Overall	4.7 (3.0–7.0)	5.7 (3.6–8.0)	0.46
Wrong box (+ instead -)	0.3 (0.0–1.7)	2.0 (0.0–4.3)	0.15
Wrong box (- instead +)	2.0 (0.0–2.7)	2.0 (0.7–2.7)	1.0
Box > 50% instead < 50%	1.7 (0.3–2.7)	1.0 (0.0–2.0)	0.35
Box < 50% instead > 50%	0.0 (0.0.– 1.0)	0.0 (0.0–1.0)	-
**Information tool B– 3D printed models** (all cases)			
Overall	4.0 (1.3–6.0)	5.0 (3.0–6.0)	0.29
Wrong box (+ instead -)	1.0 (0.0–2.0)	1.0 (0.0–2.7)	1.00
Wrong box (- instead +)	2.0 (0.0–2.3)	1.0 (0.0–4.0)	0.14
Box > 50% instead < 50%	0.3 (0.0–1.7)	0.3 (0.0–1.0)	1.00
Box < 50% instead > 50%	0.0 (0.0–1.0)	0.0 (0.0–1.0)	-
**Information tool C—MRI presentations in MDT** (all cases)			
Overall	4.3 (2.7–5.7)	5.3 (2.0–8.3)	0.45
Wrong box (+ instead -)	0.3 (0.0–1.3)	1.7 (0.0–1.3)	0.12
Wrong box (- instead +)	1.3 (0.0–2.3)	2.0 (0.0–3.3)	0.26
Box > 50% instead < 50%	0.7 (0.0–2.7)	0.3 (0.0–1.7)	0.38
Box < 50% instead > 50%	1.0 (0.0–1.0)	0.0 (0.0–1.0)	<0.001
**B2** **Minor mistakes sub-classified by the five different views in pictures 1–3 of the prostate template**			
**Information tool A—Written MRI reports** (all cases)			
Side (sagittal) view	2.0 (1.0–2.0)	1.0 (1.0–3.0)	0.17
Ventral (coronal) view	2.0 (1.0–3.0)	3.0 (2.0–4.0)	0.05
Transverse (axial) view A	0.0 (0.0–2.0)	0.0 (0.0–2.0)	-
Transverse (axial) view B	2.0 (0.0–3.0)	2.0 (1.0–2.0)	1.0
Transverse (axial) view C	0.0 (0.0–1.0)	0.0 (0.0–1.0)	-
**Information tool B– 3D printed models** (all cases)			
Side (sagittal) view	1.0 (0.0–1.0)	1.0 (1.0–2.0)	1.0
Ventral (coronal) view	2.0 (1.0–3.0)	2.0 (1.0–4.0)	1.0
Transverse (axial) view A	0.0 (0.0–1.0)	0.0 (0.0–0.0)	-
Transverse (axial) view B	2.0 (0.0–2.0)	0.0 (0.0–1.0)	0.008
Transverse (axial) view C	0.0 (0.0–1.0)	0.0 (0.0–0.0)	-
**Information tool C—MRI presentations in MDT** (all cases)			
Side (sagittal) view	1.0 (1.0–2.0)	1.0 (0.0–2.0)	1.0
Ventral (coronal) view	2.0 (1.0–3.0)	2.0 (1.0–5.0)	1.0
Transverse (axial) view	0.0 (0.0–1.0)	0.0 (0.0–1.0)	-
Transverse (axial) view B	1.0 (0.0–2.0)	1.0 (0.0–2.0)	1.0
Transverse (axial) view C	0.0 (0.0–0.0)	0.0 (0.0–1.0)	-

The rates of major mistakes in the ventral (coronal) view (decision between right and left location of the tumor) were low in both groups. Though, it was also over 0% in the 3D printed prostate models (7.4%) and in MRI presentations in MDT (3.7%) in the expert group. Notably, the rate was 0% for experts and medical students in the written MRI reports.

Minor mistakes were not significantly higher in the medical student group compared to the experts, but the students showed a trend towards a slightly higher overall minor mistake score in all of the three information tools (Table 4B1).

## Discussion

From previous MRI studies, we have learnt that knowledge of the precise three-dimensional location of a prostate cancer and its proximity to the prostate capsule and neurovascular bundle has implications for surgical therapeutic strategies, which can influence the functional or oncological outcome [1–5].

In recent years, the application of the three-dimensional (3D)-printing technology has been rapidly expanding throughout the medical field and in urological practice [16–18]. 3D printed models are used in bio printing (3D printing of biologically based materials, e.g. bone or cartilage), printed surgical equipment, preoperative planning, and surgical simulation and education. Although several studies have suggested that 3D printed models and materials may be useful tools in urological practice, only few studies are available that demonstrated the benefit in clinical outcomes. The use of customized, patient-specific printed 3D models of the prostate and cancer lesions to aid in prostate cancer surgery is even limited to only preclinical proof of concept studies [6,7]. 3D printed models are accepted as a feasible adjunct for robot-assisted radical prostatectomies [8]. However, formal evaluation of the effectiveness of 3D models in improving oncological and functional outcomes is required.

The 3D-printing technology continues to advance rapidly and promises to play an increasingly important role in the field of urology. However, the impact of 3D printed models of the prostate in the preoperative planning, surgical education, and performing of a radical prostatectomy to treat prostate cancer is currently unknown. Also currently unknown is the optimal way to transfer information of the precise location of prostate cancer (PCA) to medical professionals of different expertise utilizing various information tools, including 3D printed prostate models. MRI written reports and multidisciplinary team meetings (MDT) presenting two-dimensional images obtained from magnetic resonance imaging (MRI) are the current ‘gold standard’ and recommended by the EAU (European Association of Urology) to orientate healthcare professionals for surgical planning [19,20].

Thus, we investigated the question of how good and how reliable the exact location of a single prostate cancer lesion can get mediated to surgical experts in PCA or to inexperienced medical students by MRI written reports, MR pictures presented in MDT, or by patient-specific 3D printed prostate models, and compared the performances between the two groups of different expertise. Hence, we used a prostate template and a specifically developed scoring system to investigate the accuracy of how the location of prostate cancer lesions taken from the three different information sources were imparted to the prostate template.

Having proven construct validity of our self-designed scoring system, we could show that within the expert group, no statistically significant difference was seen concerning the overall scoring between the written MRI reports, the 3D printed prostate models, and the MRI presentations in MDT. Experts scored better in all three information tools compared to the student group. The difference was greatest after MDT information. Correspondingly, the rate of major mistakes (e.g. defined by wrong right-left and anterior-posterior plotting of the tumor lesions or plotting in wrong quadrants in the transverse views of the prostate diagram) was higher and the rate of complete major accuracy (rate of 0% major mistakes) was lower in the student group in all three information tools. Although generally inexperienced, the medical students performed best with the written reports in terms of overall scoring as well as in terms of rates of major mistakes and complete major accuracy.

The experts’ incidence of major mistakes and of major accuracy were equally high in the written MRI reports and MRI presentations in MDT, both being well established information tools. Interpretation of prostate cancer position was comparatively worse with the 3D printed prostate models, indicating a learning curve in a newly introduced source of information. However, the experts still performed better with the 3D printed prostate models compared to the students in all measured major outcome parameters, further indicating the likely effect was due to a learning curve.

Increased surgical experience has been shown to lower the complication rates of radical prostatectomies and improved cancer cure [21–23]. Lower rates of positive surgical margins for high-volume surgeons suggest that experience and careful attention to surgical details, adjusted for the characteristics of the cancer being treated, can improve cancer control with radical prostatectomy [19,24]. The impact of expertise on performance has also been studied under the generic term of cognitive psychology in different fields. It is supposed that experts base their superiority on acquired cognitive and behavioral strategies, e.g. high-level structures like schemata, and on their capability to process information holistically. The experts’ high performance in medical questions, like accurate imaging interpretation, can be further explained by a high level of perceptual skills (ability and experience to detect the abnormality) and decision skills (interpretation of the findings) [13,14].

The superior pattern recognition skills of the experts most likely explain the expert group´s significantly better performance in the MRI presentations in MDT compared to the medical students in our study. Prostate MRI understanding and interpretation, even when demonstrated by an experienced radiologist, is a complex and challenging task, not only for non-radiologists. There is evidence that novice radiologists have a learning curve of at least 50 examinations until reaching a stable fraction of correct MRI interpretations, even when receiving feedback by expert-radiologists [9,25], indicating the difficulties medical students might have to impart information received from MRI presentations in MDT. In addition, MRI presentations in MDT transfer audio-visual information, representing a highly complex information source. When the two senses both provide information about exactly the same object, combining the signals from each modality can enhance the accuracy of the resulting percept [26]. The experts are more able to take advantage of bi-sensory information tools like MRI presentations in MDT due to their superior recognition skills, whereas students likely fail to benefit as much from a visual-auditory information tool due to the complexity of MRI interpretation. Thus, it is extremely important in daily practice to perform MRI presentations in MDT in a highly structured, standardized, and reproducible way to ensure that the reported information is correctly and easily understood, not only by experts in this field [9].

MRI written reports aim to give readers the chance to process information visually in a structured and highly standardized way [10]. The written reports provide repetitious accuracy and clear information about the tumor location (in simple terms e.g. right/left or anterior/posterior or apical/basal) and tumor size with less complexity. This kind of information source seems to help inexperienced professionals to reliably take the relevant information and reproduce it. The written reports also describe the position of the prostate cancer in terms that can be directly related to the terminology on the prostate template. Which might be an explanation for the better performance of the student group with the written MRI reports in our study.

Relating to the overall scoring result, we could not show any superiority or inferiority of the 3D printed prostate models in comparison to the written MRI reports or MRI presentations in MDT in the expert group. Rather, the rates of major mistakes were nearly twice as high compared to the written reports and 5-fold higher compared to the MRI presentations in MDT. Despite the experts’ 13.3% better scoring with the 3D printed prostate models compared to the student group, the mistake rate shows that experts can struggle with this new source of information tool in comparison to those tools familiar to them and likely indicates a learning curve.

Although the students’ rate of major mistakes with the 3D printed prostate models was 1.7-fold higher and the rate of complete major accuracy was 2.1-fold lower compared to the written MRI reports, this difference resulted in only a 2.3% lower overall scoring with the 3D models compared to the written reports in the medical students group. On the other hand, the students’ overall scoring with the 3D printed models was 14.3% higher compared to the MRI presentation in MDT. This result suggests that 3D printed prostate models might be easier to understand for inexperienced trainees in comparison to MRI presentations in MDT.

Unique additional contributions of the 3D printed model are its tactile perception, as well as the visual, and the opportunity to freely turn the 3D structure and view it from different directions. Hence, this unlimited mobility increases the risk of disorientation. However, Knoedler et al. [27] recently investigated the anatomic understanding of kidney tumors of trainees, comparing 3D printed kidney models with CT (computed tomography) imaging, and showed improved accuracy of nephrometry scores achieved from the models.

Likewise, Atalay et al. investigated the impact of 3D printed pelvicaliceal system models on residents’ understanding of the pelvicaliceal system anatomy before percutaneous nephrolithotripsy surgery and recorded improved understanding of the human renal collecting system, which positively influenced surgical planning by the residents [28].

We point out that our study has limitations, which could influence the research results. The experts were already familiar with the prostate template from their daily clinical practice prior to the study. This circumstance might have been an advantage to them in drawing the exact tumor locations. In contrast, the medical student group as well as the expert group showed a learning curve with statistically significant better overall scoring in the last three plotted prostate templates compared to the first three templates (experts: 25.7 [24.0–27.0] vs. 23.0 [17.3–26.0], p = 0.03; students: 21.3 [16.0–24.0] vs. 17.3 [13.3–22.0], p = 0.06). To help avoid the effect of a learning effect over time, we used the 3x3 Latin square to prevent biases. Furthermore, our scoring system was validated by using parts of the study data, as explained in the materials and methods section. This form of construct validation for new measures is well-established and scientifically accepted [12]; however, in order to ensure its generalizability, we recommend that the construct validity of our scoring system should be further assessed with data from other populations. In addition, we like to acknowledge that individual differences in the personal ability to transfer the exact tumor location from different information tools into a 2-dimensional template (cognition) might have influenced the participants´ performances. This might have biased the results due to the limited number of study participants.

## Conclusions

In summary, overall construct validation of the scoring system and information tools was achieved, with experts showing more accurate plotting of prostate cancer location than medical students, who had more difficulty with understanding the exact tumor location, especially in the MDT setting. However, both written reports and 3D printed prostate models orientated medical students better compared to MRI presentations in MDT. This indicates that the 3D printed models might be easier to understand than the current gold standard MDT conferences. Therefore, 3D models may have a role in orientating less experienced individuals. The impact of 3D printed prostate models in surgical planning, guidance for orientation of less experienced surgeons, treatment, and subsequent outcomes of prostate cancer needs to be validated in future studies.

## Supporting information

S1 Table Raw data. (XLS)

## References

[1] Park BH, Jeon HG, Jeong BC, Seo SI, Lee HM, Choi HY, et al Influence of magnetic resonance imaging in the decision to preserve or resect neurovascular bundles at robotic assisted laparoscopic radical prostatectomy. J Urol. 2014 7;192(1):82–8. doi: 10.1016/j.juro.2014.01.005 24440235 10.1016/j.juro.2014.01.005

[2] McClure TD, Margolis DJA, Reiter RE, Sayre JW, Thomas MA, Nagarajan R, et al Use of MR imaging to determine preservation of the neurovascular bundles at robotic-assisted laparoscopic prostatectomy. Radiology. 2012 3;262(3):874–83. doi: 10.1148/radiol.11103504 22274837 10.1148/radiol.11103504

[3] Hricak H, Wang L, Wei DC, Coakley FV, Akin O, Reuter VE, et al The role of preoperative endorectal magnetic resonance imaging in the decision regarding whether to preserve or resect neurovascular bundles during radical retropubic prostatectomy. Cancer. 2004 6 15;100(12):2655–63. doi: 10.1002/cncr.20319 15197809 10.1002/cncr.20319

[4] Finley DS, Margolis D, Raman SS, Ellingson BM, Natarajan S, Tan N, et al Fine-tuning robot-assisted radical prostatectomy planning with MRI. Urol Oncol. 2013 8;31(6):766–75. doi: 10.1016/j.urolonc.2011.07.013 21906964 10.1016/j.urolonc.2011.07.013

[5] Tan N, Margolis DJA, McClure TD, Thomas A, Finley DS, Reiter RE, et al Radical prostatectomy: value of prostate MRI in surgical planning. Abdom Imaging. 2012 8;37(4):664–74. doi: 10.1007/s00261-011-9805-y 21993567 10.1007/s00261-011-9805-y

[6] Shin T, Ukimura O, Gill IS. Three-dimensional Printed Model of Prostate Anatomy and Targeted Biopsy-proven Index Tumor to Facilitate Nerve-sparing Prostatectomy. Eur Urol. 2016 2;69(2):377–9. doi: 10.1016/j.eururo.2015.09.024 26431913 10.1016/j.eururo.2015.09.024 PMC9084292

[7] Ukimura O, Aron M, Nakamoto M, Shoji S, de Abreu ALC, Matsugasumi T, et al Three-dimensional surgical navigation model with TilePro display during robot-assisted radical prostatectomy. J Endourol. 2014 6;28(6):625–30. doi: 10.1089/end.2013.0749 24450285 10.1089/end.2013.0749

[8] Porpiglia F, Bertolo R, Checcucci E, Amparore D, Autorino R, Dasgupta P, et al Development and validation of 3D printed virtual models for robot-assisted radical prostatectomy and partial nephrectomy: urologists’ and patients’ perception. World J Urol. 2017 11 10; 10.1007/s00345-017-2126-1 29127451

[9] Puech P, Randazzo M, Ouzzane A, Gaillard V, Rastinehad A, Lemaitre L, et al How are we going to train a generation of radiologists (and urologists) to read prostate MRI? Curr Opin Urol. 2015 11;25(6):522–35. doi: 10.1097/MOU.0000000000000217 26375060 10.1097/MOU.0000000000000217

[10] Barentsz JO, Richenberg J, Clements R, Choyke P, Verma S, Villeirs G, et al ESUR prostate MR guidelines 2012. Eur Radiol [Internet]. 2012 4 [cited 2017 Feb 6];22(4):746–57. Available from: http://www.ncbi.nlm.nih.gov/pmc/articles/PMC3297750/ doi: 10.1007/s00330-011-2377-y 22322308 10.1007/s00330-011-2377-y PMC3297750

[11] Barentsz JO, Weinreb JC, Verma S, Thoeny HC, Tempany CM, Shtern F, et al Synopsis of the PI-RADS v2 Guidelines for Multiparametric Prostate Magnetic Resonance Imaging and Recommendations for Use. Eur Urol. 2016 1;69(1):41–9. doi: 10.1016/j.eururo.2015.08.038 26361169 10.1016/j.eururo.2015.08.038 PMC6364687

[12] Cronbach LJ, Meehl PE. Construct validity in psychological tests. Psychol Bull. 1955 7;52(4):281–302. 13245896 10.1037/h0040957

[13] Wood G, Batt J, Appelboam A, Harris A, Wilson MR. Exploring the impact of expertise, clinical history, and visual search on electrocardiogram interpretation. Med Decis Mak Int J Soc Med Decis Mak. 2014 1;34(1):75–83. 10.1177/0272989X13492016 23811761

[14] Wood G, Knapp KM, Rock B, Cousens C, Roobottom C, Wilson MR. Visual expertise in detecting and diagnosing skeletal fractures. Skeletal Radiol. 2013 2;42(2):165–72. doi: 10.1007/s00256-012-1503-5 22940835 10.1007/s00256-012-1503-5

[15] Parente PM, Santos Silva J. Quantile regression with clustered data. J Econom Methods [Internet]. 2016 [cited 2017 Feb 20];5(1):1–15. Available from: https://www.degruyter.com/view/j/jem.2016.5.issue-1/jem-2014-0011/jem-2014-0011.xml

[16] Youssef RF, Spradling K, Yoon R, Dolan B, Chamberlin J, Okhunov Z, et al Applications of three-dimensional printing technology in urological practice. BJU Int. 2015 11;116(5):697–702. doi: 10.1111/bju.13183 26010346 10.1111/bju.13183

[17] Powers MK, Lee BR, Silberstein J. Three-dimensional printing of surgical anatomy. Curr Opin Urol. 2016 5;26(3):283–8. doi: 10.1097/MOU.0000000000000274 26825651 10.1097/MOU.0000000000000274

[18] Tack P, Victor J, Gemmel P, Annemans L. 3D-printing techniques in a medical setting: a systematic literature review. Biomed Eng Online. 2016 10 21;15(1):115 doi: 10.1186/s12938-016-0236-4 27769304 10.1186/s12938-016-0236-4 PMC5073919

[19] Professionals S-O. Prostate Cancer [Internet]. Uroweb 2014 [cited 2017 Feb 5]. http://uroweb.org/guideline/prostate-cancer/#6

[20] Mottet N, Bellmunt J, Bolla M, Briers E, Cumberbatch MG, De Santis M, et al EAU-ESTRO-SIOG Guidelines on Prostate Cancer. Part 1: Screening, Diagnosis, and Local Treatment with Curative Intent. Eur Urol. 2017 4;71(4):618–29. doi: 10.1016/j.eururo.2016.08.003 27568654 10.1016/j.eururo.2016.08.003

[21] Augustin H, Hammerer P, Graefen M, Palisaar J, Noldus J, Fernandez S, et al Intraoperative and perioperative morbidity of contemporary radical retropubic prostatectomy in a consecutive series of 1243 patients: results of a single center between 1999 and 2002. Eur Urol. 2003 2;43(2):113–8. 12565767 10.1016/s0302-2838(02)00495-5

[22] Lepor H, Nieder AM, Ferrandino MN. Intraoperative and postoperative complications of radical retropubic prostatectomy in a consecutive series of 1,000 cases. J Urol. 2001 11;166(5):1729–33. 11586211

[23] Potosky AL, Warren JL. Radical prostatectomy: does higher volume lead to better quality? J Natl Cancer Inst. 1999 11 17;91(22):1906–7. 10564667 10.1093/jnci/91.22.1906

[24] Vickers AJ, Savage CJ, Hruza M, Tuerk I, Koenig P, Martínez-Piñeiro L, et al The surgical learning curve for laparoscopic radical prostatectomy: a retrospective cohort study. Lancet Oncol. 2009 5;10(5):475–80. doi: 10.1016/S1470-2045(09)70079-8 19342300 10.1016/S1470-2045(09)70079-8 PMC2777762

[25] Rosenkrantz AB, Ayoola A, Hoffman D, Khasgiwala A, Prabhu V, Smereka P, et al The Learning Curve in Prostate MRI Interpretation: Self-Directed Learning Versus Continual Reader Feedback. AJR Am J Roentgenol. 2016 12 27;W1–W9. 10.2214/AJR.16.16876 28026201

[26] Bulkin DA, Groh JM. Seeing sounds: visual and auditory interactions in the brain. Curr Opin Neurobiol. 2006 8;16(4):415–9. doi: 10.1016/j.conb.2006.06.008 16837186 10.1016/j.conb.2006.06.008

[27] Knoedler M, Feibus AH, Lange A, Maddox MM, Ledet E, Thomas R, et al Individualized Physical 3-dimensional Kidney Tumor Models Constructed From 3-dimensional Printers Result in Improved Trainee Anatomic Understanding. Urology. 2015 6;85(6):1257–61. doi: 10.1016/j.urology.2015.02.053 26099870 10.1016/j.urology.2015.02.053

[28] Atalay HA, Ülker V, Alkan İ, Canat HL, Özkuvancı Ü, Altunrende F. Impact of Three-Dimensional Printed Pelvicaliceal System Models on Residents’ Understanding of Pelvicaliceal System Anatomy Before Percutaneous Nephrolithotripsy Surgery: A Pilot Study. J Endourol. 2016;30(10):1132–7. doi: 10.1089/end.2016.0307 27506462 10.1089/end.2016.0307

